# Exploring the Role of Excited States’ Degeneracy on Vibronic Coupling with Atomic-Scale Optics

**DOI:** 10.1021/acsnano.4c07136

**Published:** 2024-10-04

**Authors:** Kirill Vasilev, Sofia Canola, Fabrice Scheurer, Alex Boeglin, Fanny Lotthammer, Frédéric Chérioux, Tomáš Neuman, Guillaume Schull

**Affiliations:** † Université de Strasbourg, CNRS, IPCMS, UMR 7504, F-67000 Strasbourg, France; ‡ Institute of Physics, Czech Academy of Sciences, Cukrovarnická 10, 16200 Prague, Czech Republic; ¶ Université de Franche-Comté, CNRS, FEMTO-ST, F-25000 Besançon, France

**Keywords:** STM-induced luminescence, Zn-phthalocyanine derivatives, vibronic spectroscopy, vibronic coupling, Herzberg−Teller

## Abstract

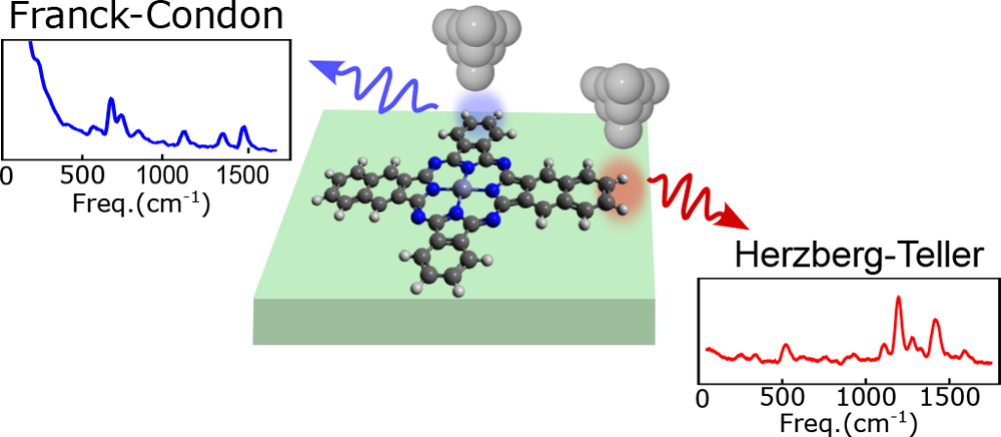

Interactions between molecular electronic and vibrational states manifest themselves in a variety of forms and have a strong impact on molecular physics and chemistry. For example, the efficiency of energy transfer between organic molecules, ubiquitous in biological systems and in organic optoelectronics, is strongly influenced by vibronic coupling. Using an approach based on scanning tunneling microscope-induced luminescence (STML), we reveal vibronic interactions in optical spectra of a series of single phthalocyanine derivative molecules featuring degenerate or near-degenerate excited states. Based on detailed theoretical simulations, we disentangle spectroscopic signatures belonging to Franck–Condon and Herzberg–Teller vibronic progressions in tip-position-resolved STML spectra, and we directly map out the vibronic coupling between the close-lying excited states of the molecules.

Vibronic phenomena involving molecular excited states play a major role in many areas of science and technology including chemistry or organic electronics.^1,2^ They can impact singlet fission which in turn affects the efficiency of organic solar cells,^3^ modulate charge separation and transport in donor–acceptor complexes,^4,5^ and influence exciton localization and coherence in organic structures.^6,7^ Their possible influence on energy transfer processes in photosynthetic complexes is the focus of many studies^8−11^ and is still heavily debated, especially because of the difficulty to properly investigate the molecular interactions and energy transfer dynamics in the complicated thermally fluctuating environment of living organisms. Unraveling these effects thus requires studying simple model systems featuring similar physical properties.^7,12,13^ In this context, it is particularly interesting to investigate vibronic interactions in molecules whose excited states are degenerate or nearly degenerate - as for porphyrines.^14−16^ Such molecules feature particularly prominent vibronic interactions strongly influencing their physics and chemistry, which in turn affect transport phenomena.

Optical vibronic spectra reflect on structural reorganization upon excitation and provide indirect information on the dynamics between the electronically excited states as in vibronic-coupling-mediated internal conversion.^17^ Subtle vibronic features in these spectra arise from different coupling mechanisms between the electronic excitation and molecular vibrations.^18^ While the Franck–Condon (FC) mechanism gives rise to signatures related to the structural reorganization of the molecule upon excitation or relaxation, phenomena such as the internal conversion are related to the Herzberg–Teller (HT) mechanism, hence to nonadiabatic coupling (NAC) between excited states. In conventional optical spectroscopy, vibronic spectra often show poor spectral resolution, because of interactions with the surrounding medium and averaging over a large number of molecules which blurs details of the vibronic features.

Here, we combine experiments and calculations to distinguish between Franck–Condon (FC) and Herzberg–Teller (HT) spectral features in a single molecule. For that we rely on scanning tunneling microscope-induced luminescence (STML) and investigate the optical spectra of single molecules with atomic-scale precision within a well-controlled environment.^19−24^ Our focus is on a series of substituted zinc(II) phthalo- and naphthalocyanine derivatives, which are considered as models of photosynthetic molecules^25^ and are relevant for many applications.^26−30^ Phthalocyanines exhibit high symmetry and possess a pair of low-lying degenerate excited states. This degeneracy is lifted in substituted molecules of lower symmetry, resulting in closely lying excited states and enhanced HT activity.^31^ These molecules therefore serve as ideal models for studying vibronic coupling phenomena at the single-molecule level. We demonstrate that using the atomic-scale spatial resolution of STML spectroscopy one can identify the molecular vibrational modes that are responsible for the interactions between close lying electronic excited states of a molecule.

## Results and Discussion

### Synthesis and STM Characterization

**Figure 1:**
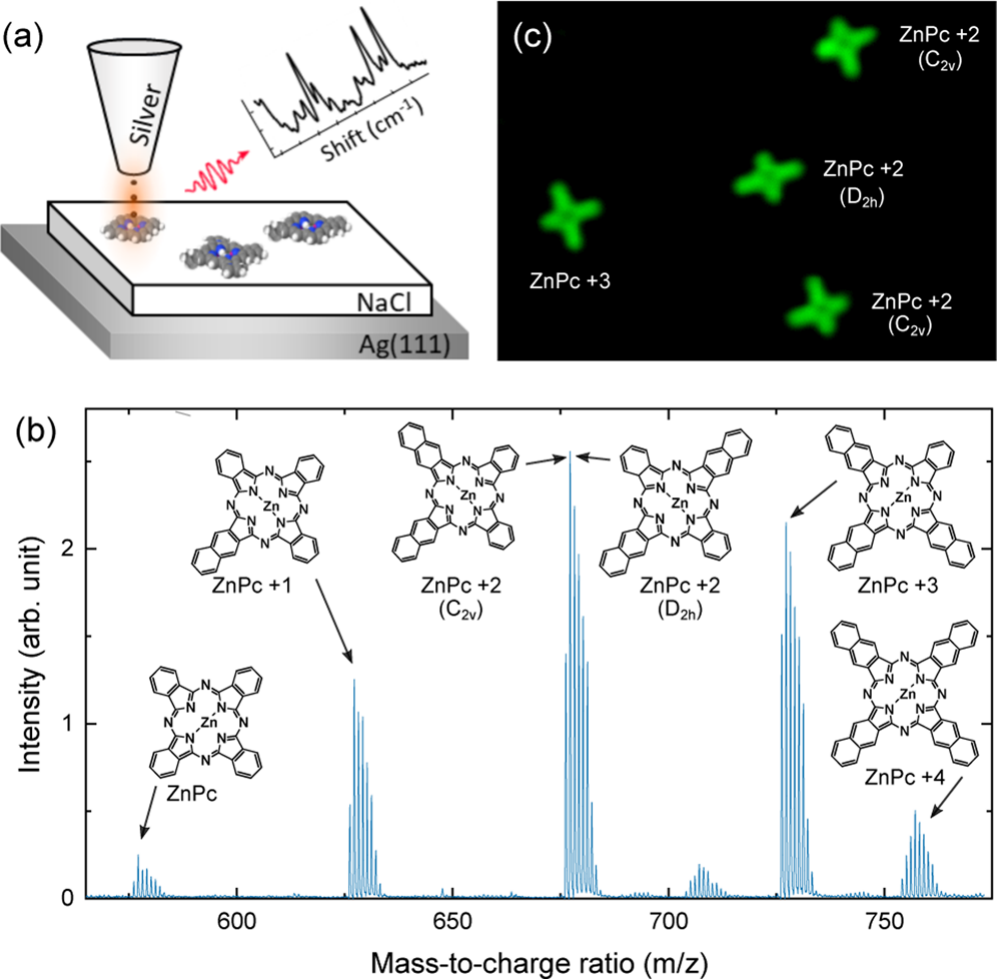
(a) Sketch of the STM-induced luminescence experiments. (b) Mass spectrum identifying the presence of the five targeted phthalocyanine derivatives. The peak at 709 *m*/*z* can be attributed to a complex of ZnPc+2 with O_2_. (c) Typical STM image (*I* = 10 pA, *V* = 0.5 V, 10 × 7 nm^2^) after sublimation of the powder on 3 ML NaCl on Ag(111).

We chose phthalocyanines as model compounds for our investigations because phthalocyanines provide a versatile and powerful molecular foundation for precise fluorescence measurements. Indeed, phthalocyanine molecules are well-known for their ability to be efficiently excited, promoting electronic transitions that generate intense fluorescence, even at the single-molecule level. In addition, the skeleton of phthalocyanine molecules can be tuned by using appropriate molecular precursors. Therefore, we synthesized a series of asymmetrically substituted zinc(II) phthalo- and naphthalocyanine derivatives. The mass spectrum of the resulting powder obtained after the complete series of Soxhlet extractions (See Supporting Information for the detailed experimental procedure) is depicted in Figure 1b. The five targeted compounds are identified by the peaks corresponding to their expected molecular masses. The short-hand notation used for naming the molecules is introduced as insets in Figure 1b. As the obtained mixture of products has a similar sublimation temperature in UHV, the deposition of all molecules occurs in a single step, simplifying strongly the experimental procedure of STM experiments.

In Figure 1c we show a typical low voltage STM image of the NaCl/Ag(111) surface after the deposition of the molecules. For this voltage condition, the images reveal patterns very close to the skeletal structure of the molecule.^32^ Hence, we unambiguously identify the two isomers of ZnPc+2, one having the two naphthalenic arms aligned (C_2*v*_), one with the arms perpendicular (D_2*h*_), and ZnPc+3.

**Figure 2:**
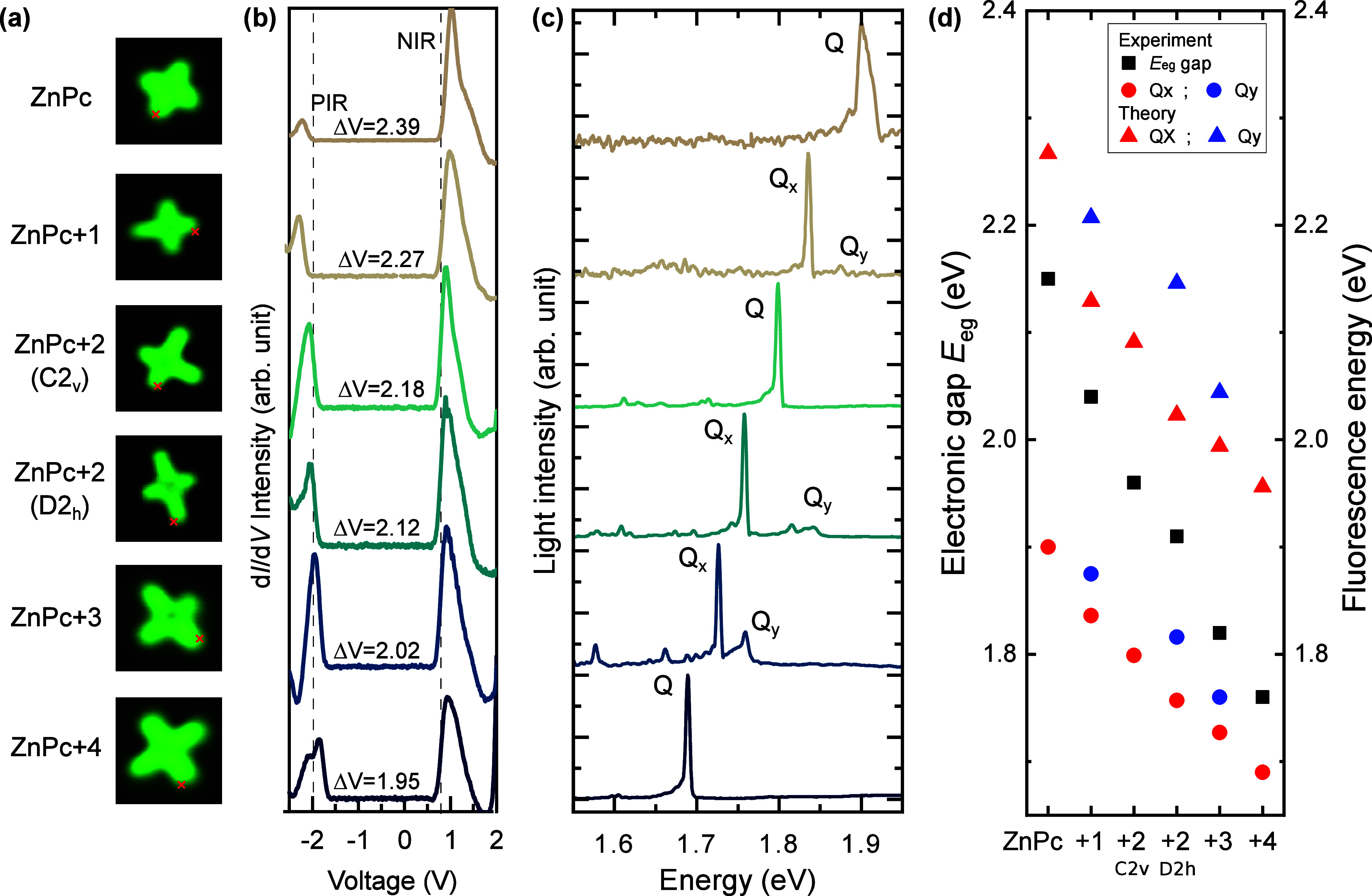
(a) STM images (*I* = 10 pA, *V* = 0.5 V, 3 × 3 nm^2^) of the different phthalocyanine derivatives adsorbed on 3 ML NaCl/Ag(111), (b) their associated conductance spectra (d*I*/d*V*) and (c) STML spectra (from top to bottom *V* = −2.5 V, *I* = 100 pA, acq. time *t* = 180 s ; *V* = −2.2 V, *I* = 200 pA, *t* = 180 s ; *V* = −2.4 V, *I* = 200 pA, *t* = 180 s ; *V* = −2.5 V, *I* = 300 pA, *t* = 120 s; *V* = −2.3 V, *I* = 100 pA, *t* = 180 s; *V* = −2.5 V, *I* = 200 pA, *t* = 120 s). Associated d*I*/d*V* maps can be found in Figure S1. The d*I*/d*V* and STML spectra were recorded for the STM tip located at the positions marked by red crosses in (a). (d) Experimental electronic gap (squares) deduced from the PIR and NIR resonances in the d*I*/d*V* spectra and fluorescence gaps (circles) estimated from the STML spectra for the different molecules. Simulated fluorescence gap (triangles) computed using TD-DFT.

STM images obtained from different NaCl islands eventually allowed for the identification of all molecules of the series (Figure 2a). This enabled us to capture their electronic and fluorescence characteristics in the form of d*I*/d*V* spectra (Figure 2b) and STML spectra (Figure 2c). In the d*I*/d*V* spectra (Figure 2b) we identify the zero-phonon energy of the positive and negative ion resonances (PIR and NIR) from which we estimate the electronic gap *E*_eg_ for each molecule. Whereas the positions of these resonances with respect to the Fermi level depend on the specific work function of the supporting substrate,^33^ the energy gap between these resonances reflects the difference between the ionization energy and the electron affinity for each molecule. We note that the shape of the d*I*/d*V* spectra is influenced by the presence of the substrate and the notable reorganization of NaCl atoms upon molecular charging.^34,35^ To estimate the electronic gap we therefore used a polaron model^36−39^ that allows us to fit the experimental peaks using a physically motivated spectral profile considering realistic values of the substrate reorganization energy.^34,35^ The details of the model are described in Section S7 of the Supporting Information. Assuming a voltage drop of 10% in the 3 ML NaCl insulating layer (estimated from a simple parallel-plate capacitor model^34^) and the model spectral profile, we deduce an electronic gap of *E*_eg_ = 2.15 eV for ZnPc, that shrinks as the molecular size (frontier-orbitals delocalization) increases in the series, and reaches *E*_eg_ = 1.76 eV for ZnPc+4 (Table 1 and Figure 2d).

**Table 1:** Experimental Electronic Gap, Emission Energies from Experimental STML Spectra and Computed Values (TD-B3LYP/6-31G*) for the First (Q_*x*_) and Second (Q_*y*_) Excited States, Experimental Exciton Binding Energy^a^

	**E**_*eg*_	**Q**_*x*_	**Q**_*y*_	**E**_bin_
	exp.	exp. (calc.)	exp. (calc.)	exp.
ZnPc	2.15	1.900 (2.267)	1.900 (2.267)	0.25
ZnPc+1	2.04	1.836 (2.129)	1.874 (2.207)	0.20
ZnPc+2/C_2*v*_	1.96	1.799 (2.091)	1.799 (2.091)	0.16
ZnPc+2/D_2*h*_	1.91	1.758 (2.023)	1.815 (2.146)	0.15
ZnPc+3	1.82	1.726 (1.994)	1.759 (2.044)	0.09
ZnPc+4	1.76	1.689 (1.956)	1.689 (1.956)	0.07

a All quantities are in eV.

### Electronic and Fluorescence STM Spectra

The d*I*/d*V* spectra also indicate how to bring the molecules to their electronically excited state with the tunneling current and thus trigger their fluorescence.^37,40^ To this end, we have to apply a negative bias to the sample to transiently populate the positively charged molecules (cations). As the PIR energy (at ≈-2 V bias) is larger than the typical fluorescence energy of the neutral phthalocyanine, the neutralization of the transiently populated cation by electron tunneling from the sample may leave the molecule in its excited state.^37,40−42^ In contrast, applying a bias corresponding to the NIR does not lead to exciton formation as the NIR energy (at ≈1 V bias) is smaller than the exciton energy.

The STML spectra (Figure 2c) are typical of phthalocyanines^25^ and reveal one main and intense emission line, as well as several peaks of lower intensities for each molecule of the series. The energy of the main peak smoothly evolves in the family of molecules, generally lowering upon increasing the molecular dimension. The higher symmetry molecules at the edges of the series (ZnPc and ZnPc+4, belonging to *D*_4*h*_ point group) display only one emission peak (Q-band) associated with a doubly degenerate electronic singlet excited state S_1_, as expected.^25^ When lowering the symmetry of the macrocycles, the Q-band splits into two peaks Q_*x*_ and Q_*y*_ associated with the two first singlet excited states S_1_ and S_2_, respectively. These peaks have variable energy separation across the series of molecules. Notably, ZnPc+2/C_2*v*_ constitutes an exception as, despite the symmetry lowering, only one peak is observed. In Table 1 and Figure 2d we report the electronic gaps (black squares) and fluorescence energy (colored dots) for the different molecules. As expected, both the electronic and optical gaps decrease as the molecular size, and consequently the π-conjugation length, increases. For a given molecule, the energy separation between these two gaps is a direct measure of the exciton binding energy. This value tends to decrease with increasing molecular size, going from ≈0.25 eV for ZnPc to ≈0.07 eV for ZnPc+4, indicating a reduced Coulomb interaction in larger molecules (Table 1 and Figure S3).

To explain these observations, we perform a series of time-dependent density functional theory (TD-DFT) calculations whose details are provided in Supporting Information. We calculate the photon emission energies for the series of molecules studied experimentally and show the results in Figure 2d and Table S1 (see also Table 1). The computed emission energies of the first and second excited states nicely reproduce the experimentally observed trend as the energy decreases with increasing molecular size. Upon symmetry lowering, the doubly degenerate excited state of the D_4*h*_ symmetry molecules splits into two excitons, whose associated transition dipoles remain oriented along the perpendicular molecular arms (Figure S4). For a more detailed analysis see Supporting Information, where we show the calculated transition densities (the oscillating electron density associated with the transition dipole moment of the excitation) of the respective excitations in the studied molecules. Interestingly, the calculations confirm that indeed for ZnPc+2/C_2*v*_ system, the Q_*x*_ and Q_*y*_ emissions occur at the same energy after geometry relaxation, and so, despite the lowered symmetry, they are degenerate.

### Vibronically Resolved STML Spectra

**Figure 3:**
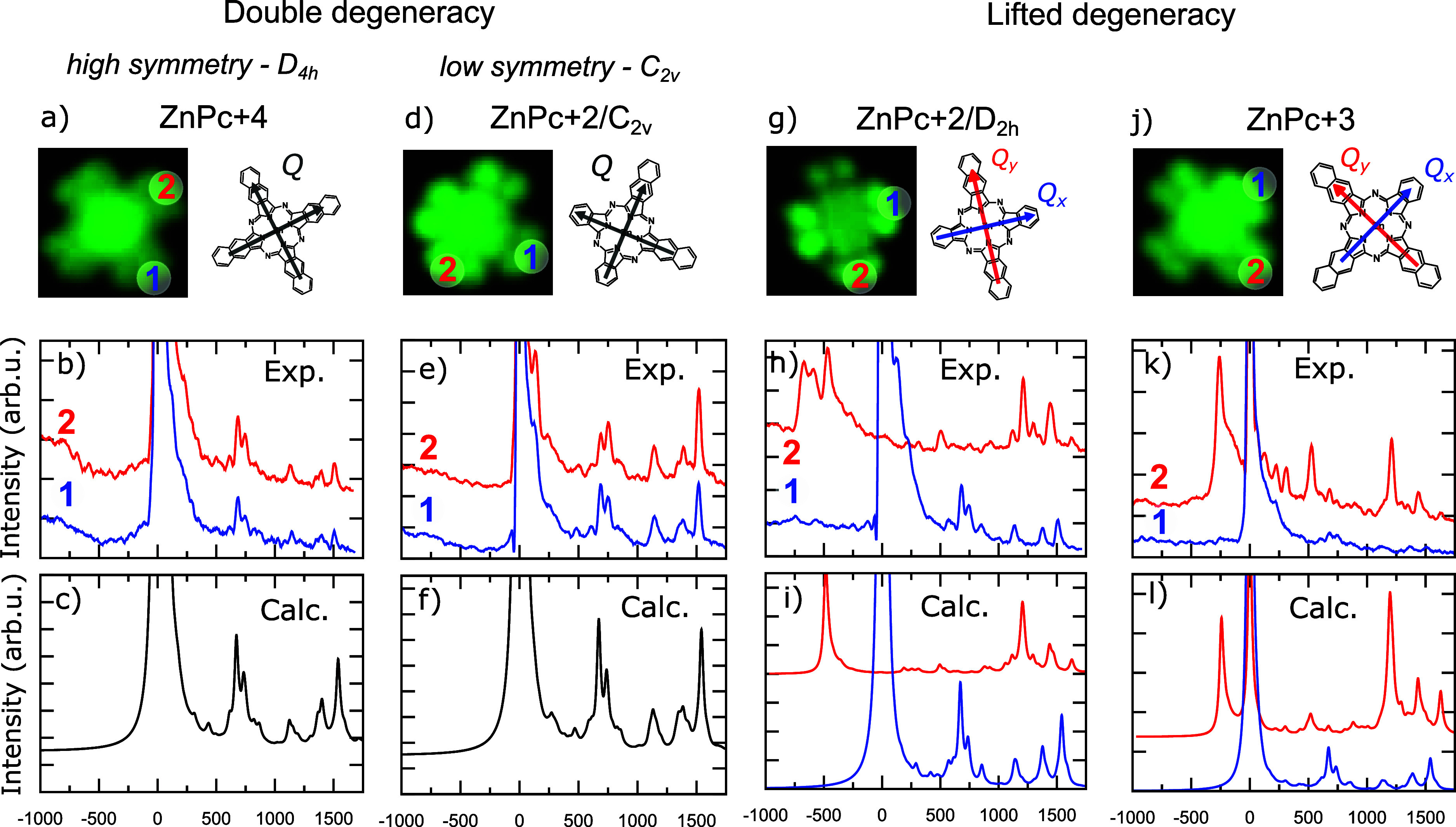
Vibronically resolved STML spectra of phthalocyanine derivatives: ZnPc+4 (a, b, c), ZnPc+2/C_2*v*_ (d, e, f), ZnPc+2/D_2*h*_ (g, h, i) and ZnPc+3 (j, k, l). STM images (*I* = 10pA, 3 × 3 nm^2^, (a) *V* = −2.5 V, (b) *V* = −2.1 V, (c) *V* = −2.5 V, (d) *V* = −2.3 V) with marked probing points and sketch of the excited states transition dipoles (a, d, g, j); experimental [b (*V* = −2.5 V), e (*V* = −2.4 V), h (*V* = −2.5 V), k (*V* = −2.3 V)] and simulated (c, f, i, l) STML spectra.

Vibronically resolved STML spectra are recorded for some of the molecules in the series for two different tip positions marked in red and blue in Figure 3a,d,g,j. These specific tip positions were chosen to be close to the high-symmetry axes of the molecule and thus allow the tip to couple specifically to one of the two transition dipoles of the molecules (arrows in the inset of Figure 3a,d,g,j - see also Figure S4).

In the experimental spectra of ZnPc+4 (Figure 3b), aside from the main emission peak at the electronic emission energy (zero-phonon line ZPL - set as zero energy for reference), the main features are associated with vibration modes at ca. 750 cm^–1^ and ca. 1500 cm^–1^. Due to the high symmetry of ZnPc+4, the interaction between the tip and the doubly degenerate S_1_ state is equivalent for positions 1 and 2 (Figure 3a and S4) and hence both spectra show the same progression (Figure 3b). The comparison of the experiment with the computed vibronic spectra with TD-DFT (see Supporting Information for details) allows us to assign the spectrum as the FC vibronic progression associated with the emission of S_1_ state (Figure 3c), with no appreciable non-Condon effects, as commonly observed for ZnPc.^43^ A similar scenario is encountered for ZnPc+2/C_2*v*_ molecule that likewise shows degenerate emission for the two excited states forming the Q-band (Figure 3e) that emit via FC mechanism (Figure 3f). More details about the degeneracy of the excited states are provided in Section S6 of the Supporting Information.

Conversely, for ZnPc+2/D_2*h*_ the experimental emission spectra obtained when placing the STM tip at two different molecular arms show different features (Figure 3h). When probing at tip position 1 (shorter arm), the spectrum (Figure 3h, blue) shows a vibronic progression closely resembling that of ZnPc+4 (compare with Figure 3b). Based on this observation, and supported by calculations (Figure 3i, blue), we can once again identify the peaks of the FC progression associated with the emission from the first excited state (Q_*x*_ band). The spectrum is different when instead the STM tip is placed along the naphthalenic arm at position 2 (Figure 3h, red), revealing an intense structured band at −400 and −700 cm^–1^ (with respect to the ZPL of the black spectrum, kept as a reference) associated with the second excited state emission (Q_*y*_ band), and a modified vibronic signature at higher frequencies: the characteristic doublet at 750 cm^–1^ is not present, while a new intense feature appears at ca. 1200 cm^–1^. In this case, differently from before, the FC emission mechanism cannot explain the new features appearing in the high-frequency spectral range. As we detail below, they correspond to the HT emission associated with the Q_*x*_ band (from the first excited state S_1_) that appears in the spectrum together with the FC spectral feature of the Q_*y*_ band (from the second excited state S_2_). The simultaneous appearance of these two spectral features together in the same spectrum suggests that S_1_ and S_2_ are interacting via vibronic coupling (NAC) as it will be also confirmed by the spatially resolved emission maps (see next section). When we position the tip at position 2 on ZnPc+2/D_2*h*_, the tip plasmon efficiently couples to S_2_ and probes its intense FC component. At the same time, the vibronic emission from S_1_ via the HT mechanism carries the transition dipole moment ”borrowed” from the S_2_ zero-phonon transition and therefore also appears in the spectrum. To confirm our attribution, the experimental spectra have been simulated (see details in Supporting Information) as a weighted sum of the FC and/or HT progressions of the Q_*y*_ and Q_*x*_ transitions, respectively. The weights were derived from the efficiency of coupling of the respective transition dipole moments with the tip plasmon and the anticipated population of the S_1_ and S_2_ states (Table S2). The spectrum modeled in this way has a very good agreement with the experimental one (Figure 3i, red). Further evidence of the relevance of NAC is also suggested by the structured envelope of the Q_*y*_ peaks in the experimental spectrum (at −400 to –700 cm^–1^, Figure 3h red). This peak splitting is likely a symptom of strong vibronic coupling mediated by a low frequency normal mode. This feature is not accounted for in the simulated spectrum, since it is composed of a simple sum of uncoupled electronic states, which is appropriate for weakly coupled vibronic modes.

The tip-position dependent STML spectra of ZnPc+3 show similar features as the related ZnPc+2/D_2*h*_ system, although with some additional complexity, probably related to the lowered symmetry of the system. Also in this case, by positioning the tip in 1 (shorter arm), Q_*x*_ emission through FC mechanism is probed (Figure 3k and l, blue spectrum). On the other hand, the spectrum probed at the longer molecular arm (position 2, Figure 3k red spectrum) is similar to that of ZnPc+2/D_2*h*_ (Figure 3h, red spectrum for comparison). In addition, the ZPL of S_1_ is present in the experimental spectrum, as the tip plasmon can couple also to the S_1_ exciton due to the reduced symmetry of the molecule although with a reduced efficiency (Figure S5). Employing our model we show that the spectrum is again dominated by the HT progression of S_1_ along with the FC progression of S_2_ and S_1_ (Figure 3l red and Figure S5 for the detailed decomposition).

### Spatially Resolved Emission Maps

**Figure 4:**
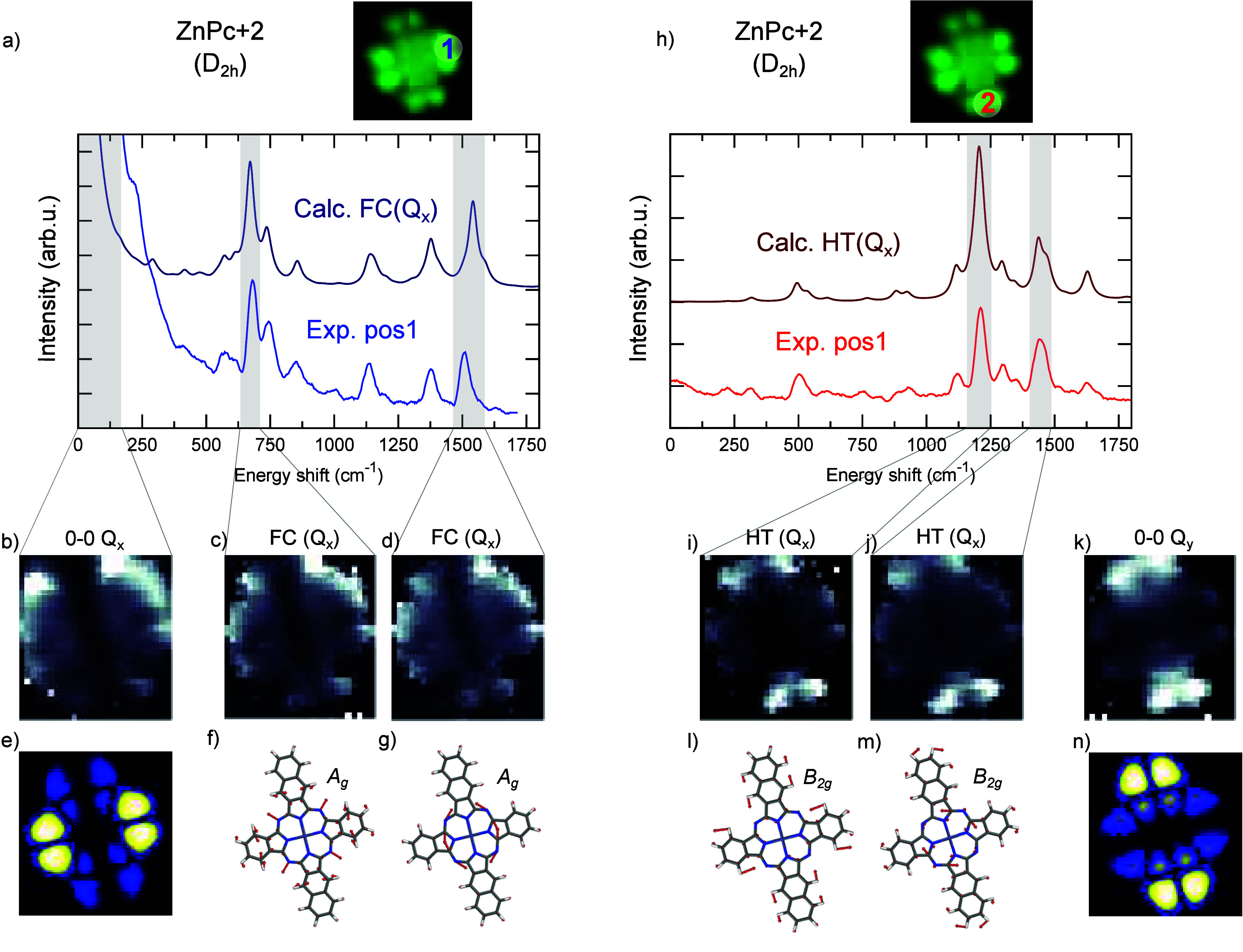
(a) STML experimental spectra (*V* = −2.5 V) probed in tip position 1 (lighter blue) and comparison to the calculated FC progression (darker blue) with (b–d) experimental emission maps (*V* = −2.5 V) of the highlighted peaks (gray shade). (e) Simulated emission map of the Q_*x*_ transition. (f, g) Calculated normal modes of vibration associated with the selected peaks (frequency 691 and 1588 cm^–1^, with symmetry label). (h) STML experimental spectra (*V* = −2.5 V) probed in tip position 2 (lighter red) and comparison to the calculated HT progression (darker red) with (i–k) associated experimental emission maps of the highlighted peaks (gray shade). (l, m) Calculated normal modes of vibration associated with the selected peaks (frequency 1239 and 1479 cm^–1^, with symmetry label). (n) Simulated emission map corresponding to the Q_*y*_ transition. The light intensity maps (2.8 × 2.6 nm^2^, *V* = −2.5 V, time per pixel = 60 s, 28 × 26 pixel grid) were recorded at constant height (i.e., open feedback loop) and normalized, pixel per pixel, by the tunnel current measured simultaneously.

The ability to scan with the STM tip across the molecule gives us a more powerful and direct way to confirm the spectral attribution. We therefore record hyper resolved fluorescence maps (HRFM) for ZnPc+2/D_2*h*_, by collecting a STML spectrum for each position of the tip with respect to the molecule. To resolve the spatial distribution of given vibronic peaks, we select the emitted light in a narrow spectral window centered around the respective vibronic energies (Figure 4a,h, shaded spectral area). By comparing the experimental spectrum recorded at specific tip positions 1 and 2 to the computed spectra, we can select peaks belonging to the FC (Figure 4a) or HT (Figure 4h) progression of the Q_*x*_ transition. The respective vibrational modes associated with the peaks are shown in Figure 4f,g,l,m (attribution in Table S3).

The experimental emission maps associated with peaks originating from the FC (at 700 and 1550 cm^–1^, Figure 4c,d) or HT (at 1200 and 1400 cm^–1^, Figure 4i,j) progression of Q_*x*_ have a markedly different shape. Alongside with the maps recorded at the energies of the vibronic peaks we show the maps at the energy of the ZPL of Q_*x*_ and Q_*y*_ transitions for comparison (Figure 4b and Figure 4k). The photon maps of the FC peaks are similar to the map of the Q_*x*_ zero-phonon line and all feature a dark node separating brighter lobes situated over the shorter benzenic arms of the molecule. On the other hand, the maps of the intense HT peaks excellently match with the one associated with the ZPL of Q_*y*_ and exhibit brighter lobes situated on the longer naphthalenic arms of the molecule. To rationalize the clearly different behaviors observed in the experiment we perform numerical simulations of the emission maps. To this end we model the interaction of the tip’s plasmon electric field and the emitting S_1_ molecular exciton, represented by its electronic transition density as computed with TD-DFT calculations. In addition, we account for the electronic pumping mechanism bringing the molecule to the excited state via the tunneling process (details of the model are in Supporting Information). For the FC-active peaks we calculate the maps directly using the transition density of the S_1_ exciton (Figure S4) and show the result in Figure 4e. This is because the FC activity affects the transition probability only by scaling the transition dipole (transition density) of the zero-phonon transition by the associated FC factors. Both the theoretical and experimental maps show a dark vertical nodal plane separating two symmetrical bright regions. The slight discrepancies in the detailed pattern between the theory and experiment can be likely attributed to inaccuracies in the theoretical normalization procedure that does not account for all the conduction channels present in the experiment. The situation is completely different for the probed intense HT active modes (Figure 4i,j). In the spirit of the HT principle, to simulate their photon maps we evaluate the numerical derivative of the transition density with respect to the vibration normal modes associated with the each peak (see Supporting Information) and use it as input to calculate the associated map (Figure 4n and Figure S6). The HT active normal modes break the molecular symmetry and mediate the NAC between S_1_ and S_2_ states. Thus, the transition density of the emitting excited state associated with the HT vibronic peak carries the evidence of this S_1_ and S_2_ states mixing (Figure S6): upon distortion along the normal mode, the transition density of S_1_ acquires a component of the transition density of S_2_ of the undistorted molecule. As we detail in Supporting Information, the derivative of the S_1_ transition density thus dominantly reflects this admixture of S_2_ components and, as a result, the spatial features of the HT-peak maps closely resemble the map of the S_2_ ZPL (Figure 4n). In this way, we can directly demonstrate S_2_ to be the excited state primarily involved in the NAC with S_1_.

Therefore, we can correlate the different patterns in HRFM to the different mechanistic origin of the signal, confirming unambiguously the spectra assignment in Figure 3. Moreover, we can offer direct access to the microscopic origin of the NAC coupling between the excited states and the electronic states that are primarily involved.

## Conclusions

In summary, we have performed a systematic experimental and theroretical study of the transport and optical properties of a series of technologically and biologically relevant phthalocyanine derivatives using STML. By analyzing subtle vibronic details of the experimentally obtained STML spectra, we have successfully revealed their HT and FC activity. Thanks to the submolecular spatial selectivity of STML, we have recorded with high spectral resolution the HT vibronic spectrum of ZnPc derivatives having nondegenerate excited states, whereas it is usually obscured by the more intense FC progression in conventional optical spectroscopy.^43^ We have concluded that the strong HT activity is a signature of NAC coupling between the two nearby lying excited states S_1_ and S_2_. Our series of ZnPc molecules allows describing the effect of a two-state degeneracy on vibronic coupling with high precision, and constitute a model system to interpret the role of NAC in similar organic structures including porphyrin-derivatives involved in energy transfer in natural photosynthetic complexes. Finally, our conclusions are reinforced by the analysis of spectrally resolved tip-position-dependent electroluminescence maps of the zero-phonon lines and intense vibronic peaks. These maps allow us to directly image the transition dipole moments of the vibronic transitions and thus verify the assignment of HT and FC peaks in the spectra. Overall, we showed that STML can be a powerful tool to study intricate excitonic-vibrational interactions with unmatched submolecular resolution.

## Methods

The STM data were obtained in ultrahigh vacuum with a low-temperature Omicron apparatus that is combined with an optical setup aiming at collecting fluorescence spectra. The light emitted at the junction is collected by a fixed lens. The colimated beam is redirected outside of the vacuum chamber and analyzed using a spectrograph coupled to a CCD camera.^44^ We used a 400 grooves/mm grating whose resolution corresponds to 2 meV (16 cm^–1^) at 1.8 eV. For the sample preparation, a crucible containing the mixed molecular powder is brought to a temperature of ≈473 K in the STM setup. The sublimed molecules are directed on a previously cleaned Ag(111) surface covered by 3 monolayers (ML) of NaCl and maintained at a temperature of 5 K. In this configuration, the molecules are sufficiently decoupled from the metal substrate to avoid luminescence quenching, while still maintaining a tunneling contact with the metal through the insulating layer. Silver tips were obtained by electrochemical etching. They were, at a later stage, gently introduced in the silver sample to adjust and optimize their plasmonic properties.

Calculations have been done employing density functional theory (DFT) and time-dependent density functional theory (TD-DFT) in the Tamm-Dancoff approximation (TDA), with B3LYP functional and 6-31G* basis set. All calculations have been performed with the Gaussian16 software.^45^ The lines have been broadened with a Lorentzian line shape with 20 cm^–1^ of half width at half-maximum. The frequencies in the spectra are rescaled by a 0.97 factor.

## Supporting Information Available

Synthetic procedure, experimental STM images and *dI*/*dV* spectra, additional details and results of calculations (excited states, vibration modes), evaluation procedure of the density of states, STML spectra and photon maps, discussion of the ZnPc+2/C_2*v*_ emission, estimate of *E*_*bin*_. (PDF)
