# Supplementary material for: Exploring the Role of Excited States’ Degeneracy on Vibronic Coupling with Atomic-Scale Optics
Source: ACS Nano. 2024 Oct 4;18(41):28052–9. doi: 10.1021/acsnano.4c07136 (PMC11483947; doi:10.1021/acsnano.4c07136)
Supplement: Supplementary file 1 — nn4c07136_si_001.pdf [file nn4c07136_si_001.pdf]

Supporting Information for Exploring the role of excited states' degeneracy on vibronic coupling with atomic-scale optics

Kirill Vasilev,[†,§] Sofia Canola,[*,‡,§] Fabrice Scheurer,[†] Alex Boeglin,[†] Fanny Lotthammer,[¶] Frédéric Chérioux,[*,¶] Tomáš Neuman,[*,‡] and Guillaume Schull[*,†]

†*Université de Strasbourg, CNRS, IPCMS, UMR 7504, F-67000 Strasbourg, France.*

‡*Institute of Physics, Czech Academy of Sciences, Cukrovarnická 10, 16200 Prague, Czech Republic.*

¶*Université de Franche-Comté, CNRS, FEMTO-ST, F-25000 Besançon, France.*

§*These authors contributed equally to this paper.*

E-mail: canola@fzu.cz; frederic.cherioux@femto-st.fr; neuman@fzu.cz; guillaume.schull@ipcms.unistra.fr

S1 Synthesis

Phthalonitrile, 2,3-dicyanonaphthalene, and $ZnBr_2$ were procured from TCI Chemicals and utilized as received without any further purification. Acetone, toluene, dichloromethane, diethyl ether, and ethanol were obtained from VWR International. Sample analysis was performed using a MicroFlex LRF instrument from Bruker Daltonics of the MALDI-TOF type. The analyses were conducted in positive mode across an m/z range from 60 to 3500, employing MALDI ionization. Data acquisition and processing were carried out using the FlexControl and FlexAnalysis software, respectively. Zinc(II) phthalocyanine was synthe-

sized following the procedure described for the synthesis of other metal phthalocyanines.[1] Phthalonitrile (0.014 mol, 2.51 g), 2,3-dicyanonaphthalene (0.014 mol, 1.79g), and $ZnBr_2$ (0.007 mol, 1.57 g) were heated in a sealed tube at 140°C under reduced pressure for 4 h. After cooling to room temperature, the resulting black solid was washed three times with 50 mL of hot diethyl ether. After filtration, the black solid was purified by Soxhlet extraction. The complete sequence of Soxhlet extraction comprised 150 mL of toluene (24 h), followed by 150 mL of dichloromethane (24 h), then 150 mL of ethanol (1 week), and finally 150 mL of acetone (one week). The resulting deep purple powder was washed with 50 mL of hot diethyl ether. This solid was subsequently subjected to mass spectrometry analysis.

S2 DFT and TD-DFT calculations.

Calculations have been done employing density functional theory (DFT) and time-dependent density functional theory (TD-DFT) in the Tamm-Dancoff approximation (TDA), with B3LYP functional and 6-31G* basis set. All calculations have been performed with the Gaussian16 software.[2] The molecular structures have been optimized in the ground state (S_0) and the first (S_1) and second (S_2) vertical excited states have been computed and subsequently relaxed to their respective minima. For the simulation of the vibronic spectra, the frequencies and normal modes of the relaxed S_0, S_1 and S_2 states have been computed with B3LYP and TD-B3LYP method (except ZnPc+3 S_2 state at TDA-B3LYP for convergence reasons). The Franck-Condon (FC) and Herzberg-Teller (HT) vibronic progressions have been calculated for S_1 and S_2 as stick spectra (grain = 1 cm^{-1}) following the implementation in refs.[2,3] The lines have been broadened with a Lorentzian lineshape with 20 cm^{-1} of half width at half maximum. The frequencies in the spectra are rescaled by a 0.97 factor.

S3 STML spectra simulation.

In the case of ZnPc+2/D_{2h} and ZnPc+3 the simulated spectra are the result of a sum of the different progressions that can be probed by a given STM tip position. The sum spectrum can be expressed as a weighted sum of the components $\mathscr{S}_x$ and $\mathscr{S}_y$:

$$S_{\mathrm{EL}}(\omega) = \mathsf{w}_x \mathscr{S}_x(\omega) + \mathsf{w}_y \mathscr{S}_y(\omega), \tag{S1}$$

where $\mathscr{S}_x$ and $\mathscr{S}_y$ are defined as follows

$$\begin{aligned} \mathscr{S}_x(\omega) &= \mathrm{FC}_{Q_x}(\omega) + n_y \mathrm{HT}_{Q_y}(\omega - \omega_0) \\ \mathscr{S}_y(\omega) &= \mathrm{HT}_{Q_x}(\omega) + n_y \mathrm{FC}_{Q_y}(\omega - \omega_0). \end{aligned} \tag{S2}$$

Each set gathers the progressions that are expected to be observed together in the spectrum, due to the probing capability of the tip at a given position. The probing capability is related with the efficient coupling to the dipole allowed transition: $\mathscr{S}_x(\mathscr{S}_y)$ is led by the dipole allowed transition to $\mathrm{Q}_x(\mathrm{Q}_y)$ which displays its FC progression together with the HT progressions of $\mathrm{Q}_y(\mathrm{Q}_x)$. The weight coefficients w_x and w_y (with $\mathsf{w}_y = 1 - \mathsf{w}_x$) represent the probability of exciting $\mathscr{S}_x$ or $\mathscr{S}_y$ in a measurement: they depend on the spatial distribution of the electronic excited states as schematized by their associated transition dipoles (so ultimately on the system symmetry) and on the experimental STM tip position. The energy onset of Q_y progression is shifted by ω_0 value, accounting for its electronic energy difference with Q_x, as estimated from the experimental spectrum (see main text). The spectra of Q_y are weighted by n_y ($n_y \leq 1$), namely the population ratio of Q_y relative to Q_x.

S4 Simulated vibronically resolved photon maps of ZnPc+2/D_{2h}

In the main text we show the calculated photon maps for the zero phonon line of the Q_x and Q_y excitons and for selected intense vibronic peaks. Here we describe the procedure employed to obtain the simulated maps.[4,5] To obtain the maps of the zero-phonon lines, we design a simple dynamical model of electronic excitation and light emission. The model contains the three most relevant states: the ground state of the molecule S_0 (state 1 in the model), the relevant excited state S_1, or S_2, (state 2 in the model), and an intermediate charged state D_0^+ (state 3 in the model) created from S_0 by inserting a hole into the highest occupied molecular orbital (HOMO). For simplicity, we do not consider the spin degeneracy of D_0^+. In the model we assume that the populations (N) of the states are governed by the rate equations:

$$\dot{N}_1 = -\gamma_T N_1 + \kappa N_2 + \gamma_{S1} N_3, \tag{S3}$$

$$\dot{N}_2 = -\kappa N_2 + \gamma_{S2} N_3, \tag{S4}$$

$$\dot{N}_3 = -\gamma_S N_3 + \gamma_T N_1, \tag{S5}$$

where $\gamma_S = \gamma_{S1} + \gamma_{S2}$, with γ_{S1} and γ_{S2} being constants expressing the substrate-mediated charging events. $\kappa(\mathbf{r}) = \kappa_{pl}(\mathbf{r}) + \kappa_{nr}$ is the total decay rate of the exciton that is composed of the tip-position-dependent plasmon-mediated decay rate $\kappa_{pl}(\mathbf{r})$ and of a non-radiative component κ_{nr} that is independent of the tip position and that arises, for example, due to the interaction of the molecular exciton with excitations of the substrate. The tip-mediated charging rate $\gamma_T(\mathbf{r})$ is treated as dependent on the tip position and is proportional to the density of states of HOMO at $z = 0.9\,\text{nm}$ above the plane of the molecule ($z = 0\,\text{nm}$). More details about γ_T are in the following section.

The intensity of the emitted light is then modelled as $\mathcal{I}(\mathbf{r}) \approx \kappa_{\text{pl}}(\mathbf{r})\langle N_2(\mathbf{r})\rangle$, with

$$\langle N_2(\mathbf{r})\rangle = \frac{\gamma_{\text{T}}(\mathbf{r})\gamma_{\text{S2}}}{\gamma_{\text{T}}(\mathbf{r})\gamma_{\text{S2}} + \kappa(\mathbf{r})[\gamma_{\text{T}}(\mathbf{r}) + \gamma_{\text{S}}]} \tag{S6}$$

being the steady-state population of the excited state.

We obtain the spatial dependency of $\kappa_{\text{pl}}(\mathbf{r})$ assuming that a single plasmonic mode couples to the exciton. In this approximation:

$$\kappa_{\text{pl}}(\mathbf{r}) \approx \frac{K_{\text{pl}}|g(\mathbf{r})|^2}{\max\{|g(\mathbf{r})|^2\}}, \tag{S7}$$

where K_{pl} is a constant and g is the plasmon-exciton coupling. To evaluate g, we first calculate the transition density ρ_{x} (ρ_{y}) of the respective excitations Q_x (Q_y) using TD-DFT and extract the numerical data in the form of a cube file using Multiwfn.[6] We calculate g as a convolution of the transition densities ρ_i with a model profile of the plasmonic quasi-static potential ϕ_{pl}:

$$\hbar g(\mathbf{r}') = \int \rho_i(\mathbf{r})\phi_{\text{pl}}(\mathbf{r} - \mathbf{r}')\mathrm{d}^3\mathbf{r}, \tag{S8}$$

where $\hbar$ is the reduced Planck constant, $\mathbf{r}'$ is the position of the tip, and $i \in \{\text{x}, \text{y}\}$. We model ϕ_{pl} as the potential generated by a pair of point charges of opposite sign but equal magnitude positioned at $z_1 = 1.5\,\text{nm}$ above and $z_2 = -2.5\,\text{nm}$ below the plane of the molecule ($z = 0\,\text{nm}$), but equal lateral position.

Finally, the experimental map is obtained as the photon intensity normalized by the tunneling current. We therefore simulate the photon maps $M(\mathbf{r})$ as:

$$M(\mathbf{r}) = \frac{\mathcal{I}(\mathbf{r})}{I(\mathbf{r})}, \tag{S9}$$

where we assume that the tunneling current $I(\mathbf{r}) \propto \gamma_{\text{T}}(\mathbf{r}) + C\max\{\gamma_{\text{T}}(\mathbf{r})\}$ is mostly propor-

tional to γ_T, but also includes a component that only weakly depends on the tip position (modelled as tip-position-independent with a current background defined by the constant $C = 0.1$, for simplicity) and arises from other non-resonant tunneling channels that are not included in our simple model.

The emission maps of the vibronic peaks of Q_x associated with the FC active modes are calculated employing ρ_x and for all peaks coincide with those recorded at the ZPL. This is because the FC principle only scales the relevant transition density by a factor determined by the overlap of the vibrational wave functions in the ground and in the excited electronic state of the molecule. We have also verified that the distortion of the molecule along the FC-active modes has only a minor effect on the distribution of the transition density and thus neglected their HT activity.

In virtue of the Herzberg-Teller principle, the emission maps of the vibronic peaks of Q_x associated with the HT active normal modes are estimated with the method of finite differences. The numeric derivative $\partial\rho_\mathrm{x}/\partial q_k$ of the electronic transition density associated to Q_x with respect to the geometrical distortion along the k-th normal mode of interest q_k is computed using the method of finite differences:

$$\frac{\partial\rho_\mathrm{x}(q)}{\partial q_k} \approx \frac{\rho_\mathrm{x}(q+\tilde{q}) - \rho_\mathrm{x}(q)}{\tilde{q}}. \tag{S10}$$

The displacement width is defined in units of quantum amplitude $\tilde{q}$ (a.u.), that depends on the frequency ω_k of each selected mode: $\tilde{q_k} = 1/\sqrt{2\omega_k}$. The adopted displacement is $1\,\tilde{q}$ for all modes, after verifying to be in linear regime of distortion. Practically, the equilibrium structure and normal modes are computed with DFT. The molecule geometry is then distorted along the selected q_k, and S_1 with the associated ρ_x are extracted. The emission maps are then simulated similarly to the ones of the ZPL and FC transitions, but the transition density ρ in Eq. S8 is replaced by its derivative.

S5 Evaluating density of states

As discussed in the previous section, the tip-mediated charging rate γ_{T} of the molecule is responsible for injecting a hole into the HOMO of the molecule. This transition rate is therefore proportional to the density of states associated with the HOMO evaluated at the position of the tip. To properly model the spatial dependence of the normalized photon intensity maps, we therefore need to evaluate the HOMO as a function of the tip position at a constant height above the molecular plane. Since we need to properly account for the spatial distribution of the orbital at a larger distance from the atoms of the molecule, Gaussian-based molecular orbitals used in the TD-DFT calculations become inadequate. We therefore resort to an implementation of DFT that evaluates the Kohn-Sham orbitals on a real-space grid as implemented in Octopus.[7] To extract the molecular orbitals we perform a DFT ground-state calculation with the local density approximation (LDA) using the Slater exchange[8,9] and modified correlation contribution[10] by Perdew and Zunger. We use the minimal box size with a radius of 10 Å around the atoms and a mesh step of 0.1 Å. LDA pseudopotentials have been used to account for the effect of core electrons in C, H, N, and Zn.

S6 Degenerate emission from the ZnPc+2/C_{2v} system

In the main text we discuss that the emission energy of the x and y oriented excitons is degenerate; a surprising observation owing to the C_{2v} symmetry of the molecule in its electronic ground state. When performing excited-state calculations in the ground-state C_{2v} geometry we indeed obtain a pair of almost degenerate excited states (Q_x and Q_y) whose transition dipole moments lie in the σ'_{v} symmetry plane and are oriented one along the σ_{v} symmetry plane of the molecule and one perpendicular to it. When calculating the emission, we relax the molecule in the excited state. At the minima of the potential energy surfaces of Q_x and Q_y, the molecule breaks its ground-state symmetry and lowers its symmetry (C_1

point group). The main deformation consists in stretching the molecule along one opposing pair of benzo- and naphthalo- arms and shrinking it along the other one. This results in a pair of equivalent excited-state geometries, depending on which pair undergoes shrinking (stretching). The resulting transition dipoles of the lowest excited state in the respective geometries are then oriented along the molecular arms and are related to each other by the reflection operation with respect to the σ_v mirror plane of the C_{2v} molecule (Fig. S4). The STML data indeed report a single optical transition, consistent with transitions involving two degenerated states.

S7 Exciton binding energy estimation E_bin

In Table 1 of the main text, we report the estimate of the exciton binding energy E_bin. This value is estimated as a difference between the electronic gap energy E_eg and the energy of the lowest radiative transition (Q_x) of each molecule. First we considered that the voltage drop between the sample molecule and the tip represents only ≈90 % of the total bias applied between the tip and the substrate. We therefore re-scale the voltage axis on the $\mathrm{d}I/\mathrm{d}V$ experimental data by a factor of 0.9.

Moreover, the $\mathrm{d}I/\mathrm{d}V$ peaks of molecules on NaCl are broadened due to the interaction of the charged molecule with the substrate atoms that is triggered upon charging the molecule and leads to the reorganization of the NaCl lattice.[11,12] As a consequence, the observed peaks can be understood as Franck-Condon vibronic transitions where phonons in the NaCl layer are created with a probability corresponding to their Franck-Condon coefficients. We therefore develop a simple theoretical framework that accounts for this Franck-Condon effect and use a profile function $S(V)$ to model the onsets of the experimental $\mathrm{d}I/\mathrm{d}V$ peaks and extract the zero-phonon transition energies eV_0. We note that in the experiment, the zero-phonon transition is not observable as, for the relatively large reorganization energy, the onset of the peaks already includes vibronic transitions where NaCl phonons are created.

Using the polaron theory,[13,14] we get for the spectral profile $S(V)$ in $\mathrm{d}I/\mathrm{d}V$:

$$S(V) \propto \mathrm{Re}\left\{\int_0^\infty e^{ieV_0t/\hbar+\int_0^\infty J(\Omega)e^{i\mathrm{sgn}(V_0)\Omega t}\mathrm{d}\Omega}e^{-ieVt/\hbar}\mathrm{d}t\right\}, \tag{S11}$$

where V_0 is the voltage corresponding to the zero-phonon transition, $e = |e|$ is the elementary charge, and $J(\Omega)$ is a continuous function of angular frequency Ω that can be linked to the reorganization energy E_R related to the rearrangement of atoms in the substrate upon charging the molecule:

$$E_\mathrm{R} = 2\int_0^\infty \hbar\Omega J(\Omega)\,\mathrm{d}\Omega. \tag{S12}$$

For relatively high values of $E_\mathrm{R} \approx 0.8\,\mathrm{eV}$ expected for the molecule on the NaCl substrate[11,12] it appears that the exact form of $J(\Omega)$ does not strongly influence the main features observed in the spectrum. We therefore use a simple spectral function of the form:

$$J(\Omega) = \eta\,\mathrm{rect}\left(\frac{\Omega - \Omega_\mathrm{min} - 0.5(\Omega_\mathrm{max} - \Omega_\mathrm{min})}{\Omega_\mathrm{max} - \Omega_\mathrm{min}}\right), \tag{S13}$$

where eV_0 is the zero-phonon energy of the transition and $\Omega_\mathrm{min} = 18\,\mathrm{meV}$ and $\Omega_\mathrm{max} = 31\,\mathrm{meV}$ are the lower and upper bound of the optical-phonon frequencies in NaCl[15,16] and $\hbar\eta = E_\mathrm{R}/(\Omega^2_\mathrm{max} - \Omega^2_\mathrm{min})$.

For completeness, we provide the fitted $\mathrm{d}I/\mathrm{d}V$ peaks in Fig. S2 corresponding to the data shown in Table 1 of the main text. The experimental data (blue lines) and the model result (red dashed lines) are both normalized to their maxima for clarity. The model nicely reproduces the onsets of the first $\mathrm{d}I/\mathrm{d}V$ peaks. The discrepancies appearing at higher $|V|$ can be attributed to the opening of other conduction channels including vibronic transitions.[14] We therefore choose the model parameters so it fits to the onsets of the experimental peaks.

S8 Tables and figures

Table S1: Computed first two electronic excited state properties: absorption and emission energies; oscillator strength of the latter.

	state	Abs./ eV	Emiss./ eV	osc.s.
**ZnPc**	S_1<br>S_2	2.300	2.267	0.623
**ZnPc+1**	S_1	2.173	2.129	0.890
	S_2	2.245	2.207	0.890
**ZnPc+2/C$_{2v}$**	S_1	2.132	2.091	0.791
	S_2	2.133	2.091	0.791
**ZnPc+2/D$_{2h}$**	S_1	2.063	2.023	0.527
	S_2	2.187	2.146	1.182
**ZnPc+3**	S_1	2.032	1.994	0.734
	S_2	2.085	2.044	1.045
**ZnPc+4**	S_1<br>S_2	1.993	1.956	0.971

Table S2: Parameters for the simulated vibronic spectra.

	w_x	w_y	n_y	$\omega_0/\mathrm{cm}^{-1}$
ZnPc+4	1.000	0.000	0.000	-
ZnPc+2/D$_{2h}$	1.000	0.000	0.025	500
ZnPc+3	0.100	0.900	0.050	250
ZnPc+2/C$_{2v}$	1.000	0.000	0.000	-

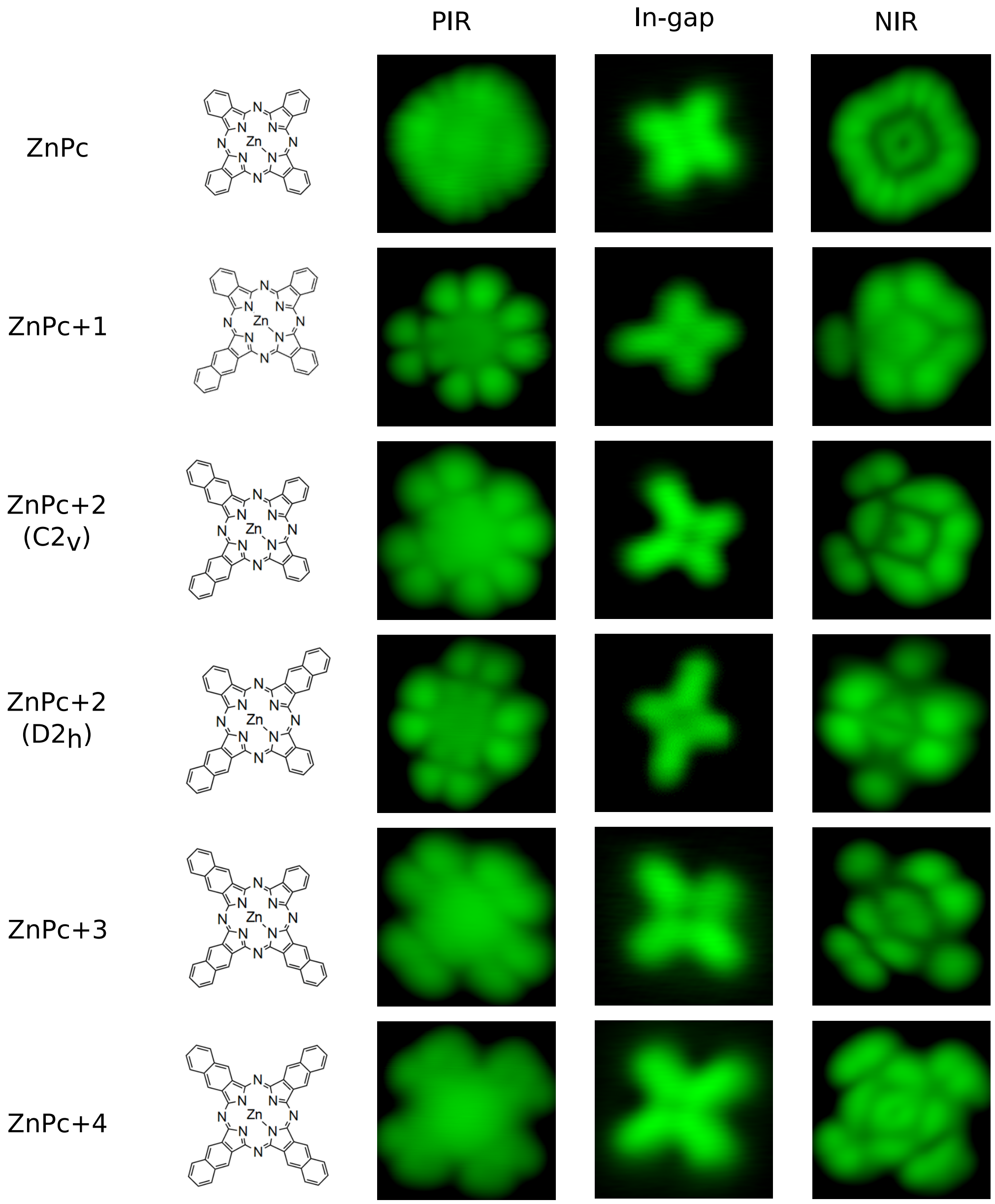

Figure S1: STM images ($I = 10\,\mathrm{pA}$, $3 \times 3\,\mathrm{nm}^2$) acquired at the energy of the positive ion resonances (PIR), in-gap ($V = 0.5\,\mathrm{V}$), and at the energy of the negative ion resonances (NIR) as deduced from $\mathrm{d}I/\mathrm{d}V$ spectra (see Fig. 2 in the main text).

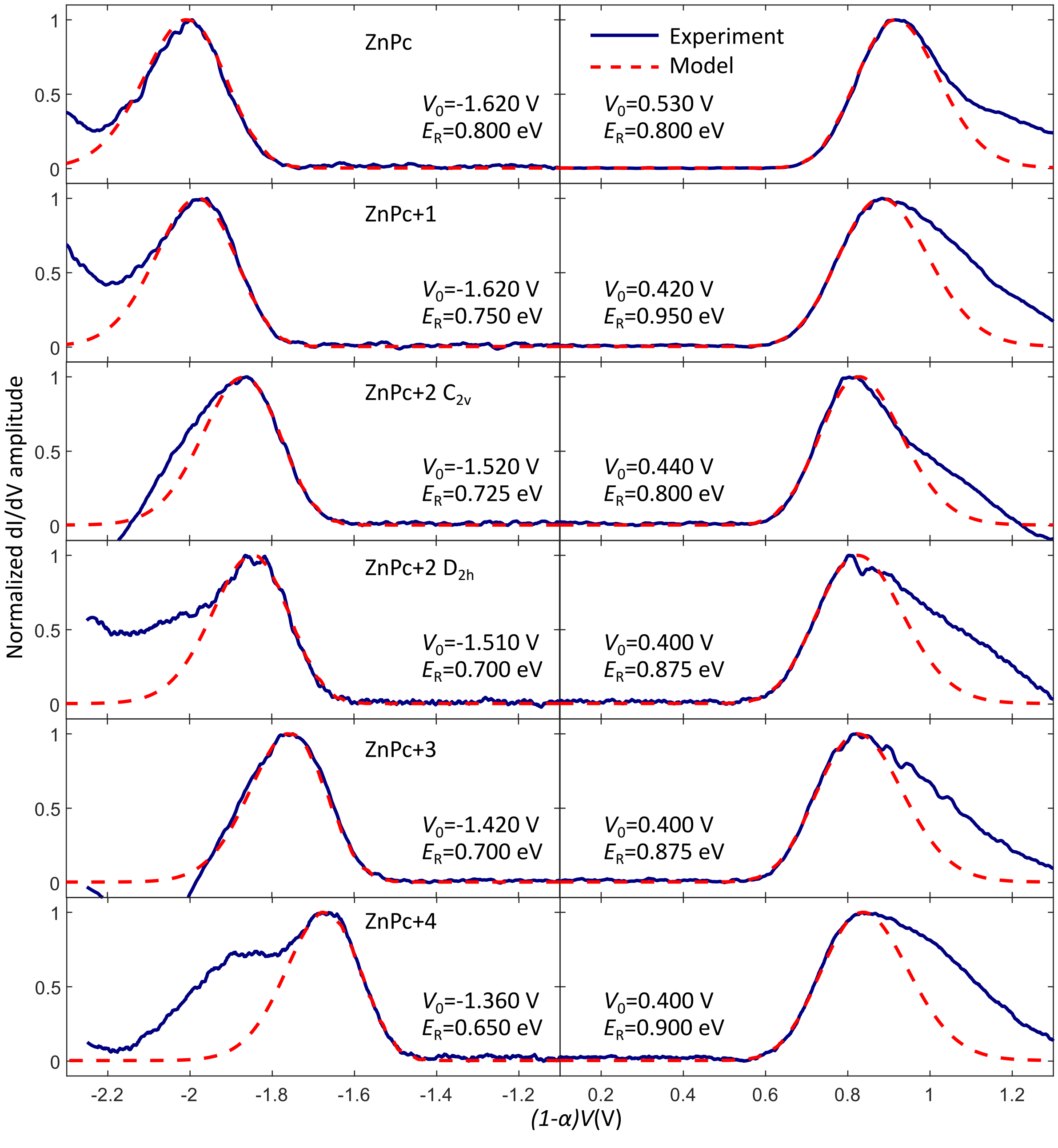

Figure S2: Fitting the model (red dashed lines) to the experimental dI/dV spectra (blue lines) for the series of molecules. The values of V_0 and E_{R} used to fit the data are included in the inset.

Table S3: Computed spectra of ZnPc+2/D_{2h}: active vibration normal mode, frequency and irreducible representation of the symmetry group.

**n.mode**	**Freq./cm^{-1}**	**symm.**
Franck-Condon active		
64	691	A_g
171	1588	A_g
70	759	A_g
152	1420	A_g
124	1170	A_g
Herzberg-Teller active		
133	1239	B_{2g}
157	1479	B_{2g}
137	1255	B_{2g}
141	1334	B_{2g}
122	1148	B_{2g}
181	1679	B_{2g}
163	1518	B_{2g}
46	508	B_{2g}

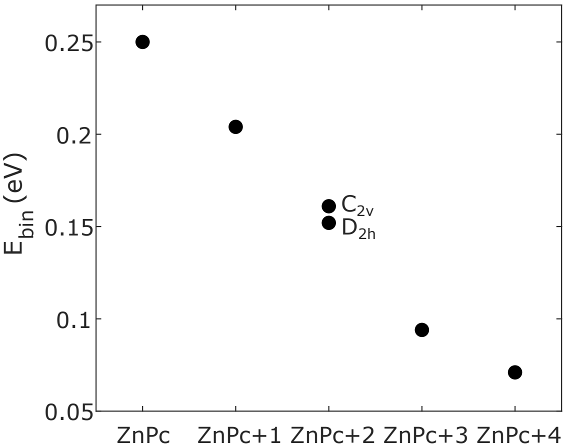

Figure S3: Estimated exciton binding energy as a function of the molecular size derived from the experimental data and the polaron model.

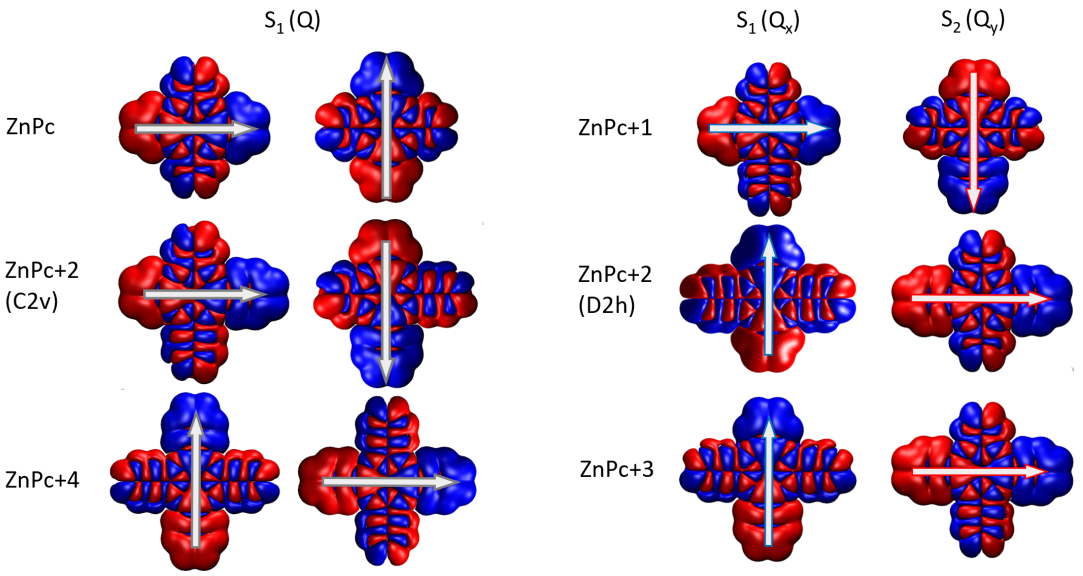

Figure S4: Computed electronic transition densities of states S_1 (Q_x) and S_2 (Q_y) and their associated transition dipole moment.

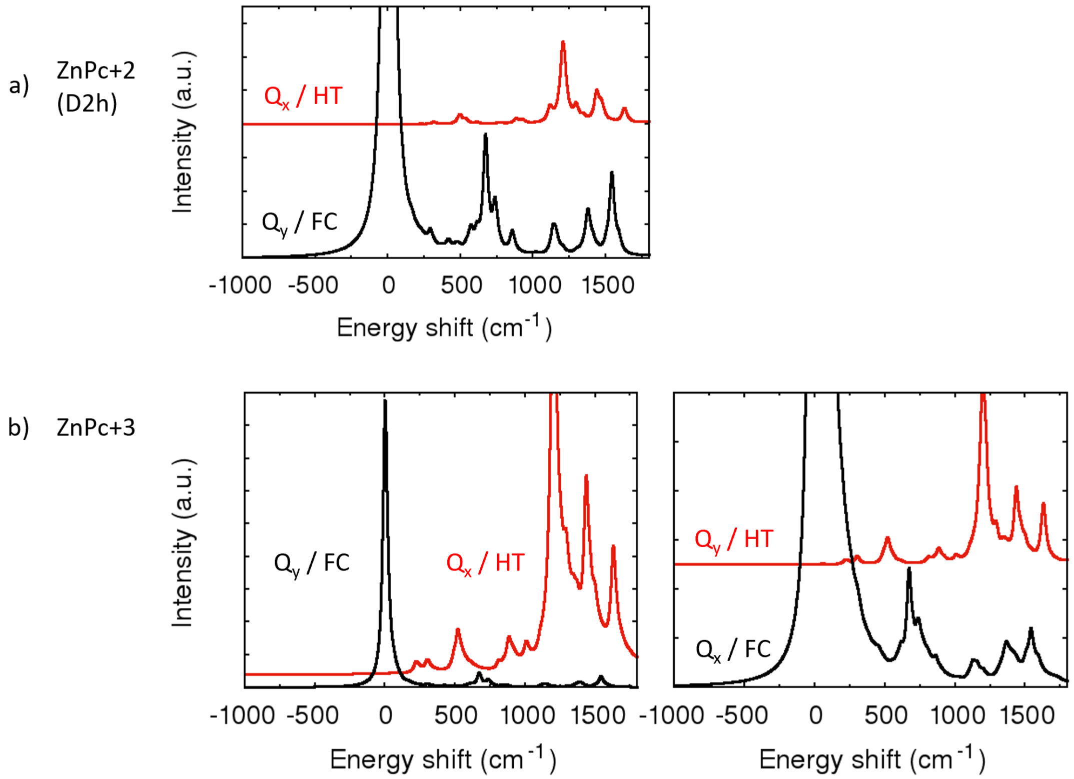

Figure S5: Computed vibronic progressions. a) ZnPc+2/D_{2h}: FC of $S_1(Q_x)$ and HT of $S_2(Q_y)$; b) ZnPc+3: FC and HT of both $S_1(Q_x)$ and $S_2(Q_y)$.

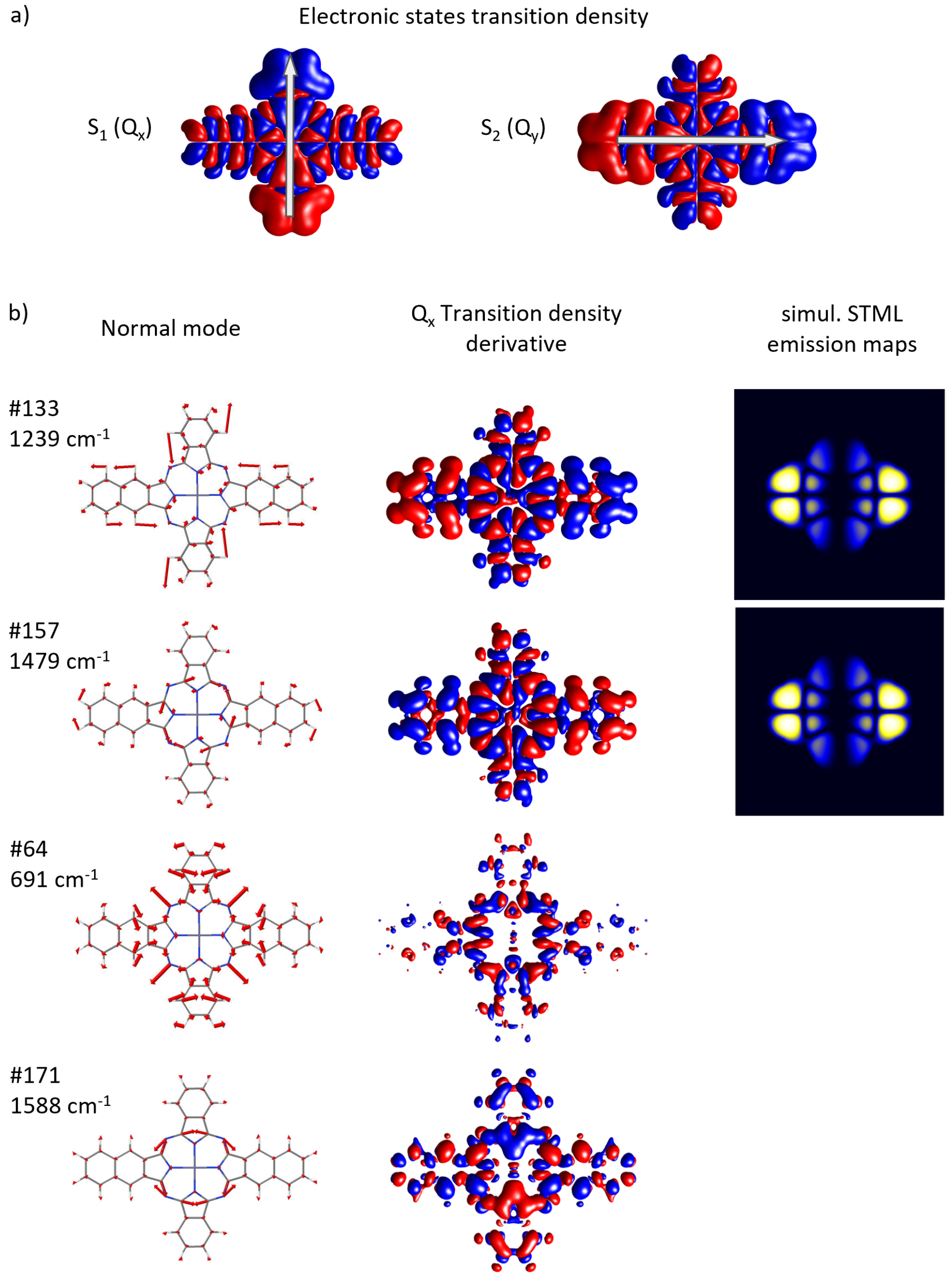

Figure S6: ZnPc+2/D$_{2h}$: a) Computed transition densities of S_1(Q$_x$) and S_2(Q$_y$) electronic state and the associated transition dipole moment (white arrow). b) Herzberg-Teller active modes (modes number 133, 157), the first derivative of the electronic transition density of S_1 excited state with respect to the associated normal mode and simulated emission maps; Franck-Condon active modes (modes number 64, 171) and the first derivative of the electronic transition density of S_1 excited state with respect to the associated normal mode.

References

(1) Lokesh, K. S.; Adriaens, A. Synthesis and characterization of tetra-substituted palladium phthalocyanine complexes. *Dyes and Pigments* **2013**, *96*, 269–277.

(2) Frisch, M. J. et al. Gaussian16 Revision C.01. 2016; Gaussian Inc. Wallingford CT.

(3) Santoro, F.; Lami, A.; Improta, R.; Bloino, J.; Barone, V. Effective method for the computation of optical spectra of large molecules at finite temperature including the Duschinsky and Herzberg-Teller effect: The Qx band of porphyrin as a case study. *The Journal of Chemical Physics* **2008**, *128*, 224311.

(4) Doppagne, B.; Neuman, T.; Soria-Martinez, R.; López, L. E. P.; Bulou, H.; Romeo, M.; Berciaud, S.; Scheurer, F.; Aizpurua, J.; Schull, G. Single-Molecule Tautomerization Tracking through Space- and Time-Resolved Fluorescence Spectroscopy. *Nat. Nanotechnol.* **2020**, *15*, 207–211.

(5) Jiang, S.; Neuman, T.; Bretel, R.; Boeglin, A.; Scheurer, F.; Le Moal, E.; Schull, G. Many-Body Description of STM-Induced Fluorescence of Charged Molecules. *Physical Review Letters* **2023**, *130*, 126202.

(6) Lu, T.; Chen, F. Multiwfn: A multifunctional wavefunction analyzer. *Journal of Computational Chemistry* **2012**, *33*, 580–592.

(7) Tancogne-Dejean, N. et al. Octopus, a computational framework for exploring light-driven phenomena and quantum dynamics in extended and finite systems. *Phys. Chem. Chem. Phys.* **2020**, *152*, 124119.

(8) Dirac, P. A. M. Note on Exchange Phenomena in the Thomas Atom. *Math. Proc. Cambridge Philos. Soc.* **1930**, *26*, 376.

(9) Slater, J. A Simplification of the Hartree-Fock Method. *Phys. Rev.* **1951**, *81*, 385.

(10) Perdew, J. P.; Zunger, A. Self-Interaction Correction to Density-Functional Approximations for Many-Electron Systems. *Phys. Rev. B* **1981**, *23*, 5048.

(11) Fatayer, S.; Schuler, B.; Steurer, W.; Scivetti, I.; Repp, J.; Gross, L.; Persson, M.; Meyer, G. Reorganization energy upon charging a single molecule on an insulator measured by atomic force microscopy. *Nature Nanotechnology* **2018**, *13*, 376–380.

(12) Hernangómez-Pérez, D.; Schlör, J.; Egger, D. A.; Patera, L. L.; Repp, J.; Evers, F. Reorganization energy and polaronic effects of pentacene on NaCl films. *Phys. Rev. B* **2020**, *102*, 115419.

(13) Mahan, G. D. *Many Particle Physics, Third Edition*; Plenum: New York, 2000.

(14) Miwa, K.; Imada, H.; Imai-Imada, M.; Kimura, K.; Galperin, M.; Kim, Y. Many-Body State Description of Single-Molecule Electroluminescence Driven by a Scanning Tunneling Microscope. *Nano Letters* **2019**, *19*, 2803–2811.

(15) Raunio, G.; Rolandson, S. Lattice Dynamics of NaCl, KCl, RbCl, and RbF. *Phys. Rev. B* **1970**, *2*, 2098–2103.

(16) Messaoudi, I. S.; Zaoui, A.; Ferhat, M. Band-gap and phonon distribution in alkali halides. *Phys. Status Solidi B Basic Res.* **2015**, *252*, 490–495.
